# Evaluation of large language models for PI-RADS score extraction from free-text prostate MRI reports: a comparative study with human readers

**DOI:** 10.3389/fonc.2026.1743096

**Published:** 2026-04-10

**Authors:** Jing Wen, Yifan Qian, Xun Chen, Yifan Cui, Jingjing Hao, Xin Yuan, Zhihan Tan, Zihao Zhou, Wenhao Meng

**Affiliations:** Department of Medical Imaging, Jiangsu Medical College, Yancheng, China

**Keywords:** diagnostic performance, large language model, mpMRI, PI-RADS, prostate cancer

## Abstract

**Objective:**

This study aimed to evaluate the ability of GPT-4o and Gemini 2.5 Pro to extract and assign PI-RADS v2.1 score from free-text prostate MRI reports, and compare their performance with human readers of varied experience.

**Methods:**

Three radiologists with differing levels of experience (resident, fellow, expert) independently reviewed the reports and assigned PI-RADS v2.1 scores. The same reports were processed through prompts with the GPT-4o and Gemini 2.5 Pro. Inter-rater agreement was evaluated using Gwet’s AC1 coefficient, and the diagnostic performance was assessed using sensitivity, specificity, and area under the receiver operating characteristic curve (AUC).

**Results:**

Inter-rater agreement between human experts was highest between the expert and fellow (Gwet’s AC1 = 0.68, 95% CI 0.61-0.75), which was significantly higher than between two LLMs (Gwet’s AC1 = 0.52, 95% CI 0.44-0.59, P = 0.004). The agreement between expert and GPT (Gwet’s AC1 = 0.42, 95% CI 0.34-0.51) was lower than between expert and Gemini (Gwet’s AC1 = 0.49, 95% CI 0.41-0.57, P = 0.17). The AUCs for resident, fellow, and expert readers were 0.81 (95% CI 0.76-0.87), 0.86 (95% CI 0.81-0.91), and 0.89 (95% CI 0.85-0.93), and for GPT and Gemini were 0.85 (95% CI 0.81-0.90) and 0.84 (95% CI 0.80-0.89), respectively.

**Conclusion:**

LLMs demonstrated promising performance in assigning PI-RADS scores from free-text prostate MRI reports, with accuracy and agreement approaching that of general radiologists; however, they are not yet ready to replace expert interpretation in high-stakes clinical settings. Nevertheless, these findings support its potential as a supplementary tool for report standardization and trainee education.

## Introduction

Prostate cancer is the second most common cancer and the fifth leading cause of cancer-related deaths among men globally (1). The mpMRI has emerged as a pivotal tool in the detection, localization, and risk stratification of csPCa, particularly in the pre-biopsy evaluation. To standardize prostate MRI interpretation, the PI-RADS was introduced by the American College of Radiology (ACR) and has since become the international benchmark for reporting prostate MRI findings (2–4). Despite the availability of structured PI-RADS templates, free-text reporting remains widespread in clinical practice due to time constraints, system limitations, and reader preference. These narrative reports, while rich in diagnostic information, often lack standardization, contributing to interpretive variability and potential diagnostic discordance (5). Moreover, the assignment of PI-RADS scores requires a nuanced understanding of MRI sequences and lesion characteristics, and interpretation accuracy can vary significantly depending on the reader’s level of training and experience (6).

In recent years, advances in artificial intelligence (AI) particularly LLMs such as ChatGPT, have shown promise in clinical natural language processing tasks (7, 8). Trained on vast corpora of internet text and fine-tuned for instruction-following behavior, LLMs exhibit strong capabilities in reading comprehension, summarization, information extraction, and medical reasoning (9–11). In radiology, LLMs are being explored for applications ranging from automatic report generation to structured data extraction, with early evidence suggesting that they can approach expert-level performance in certain contexts (12–16). However, the utility of LLMs in interpreting and extracting structured data from complex radiologic text-such as PI-RADS scoring from prostate MRI reports-has not been comprehensively evaluated. In this study, we aimed to investigate the capability of GPT-4o and Google Gemini 2.5 pro to extract and assign PI-RADS scores from free-text prostate MRI reports, then compared these LLMs with human radiologists of varied experience.

## Methods

### Study design

**Figure 1:**
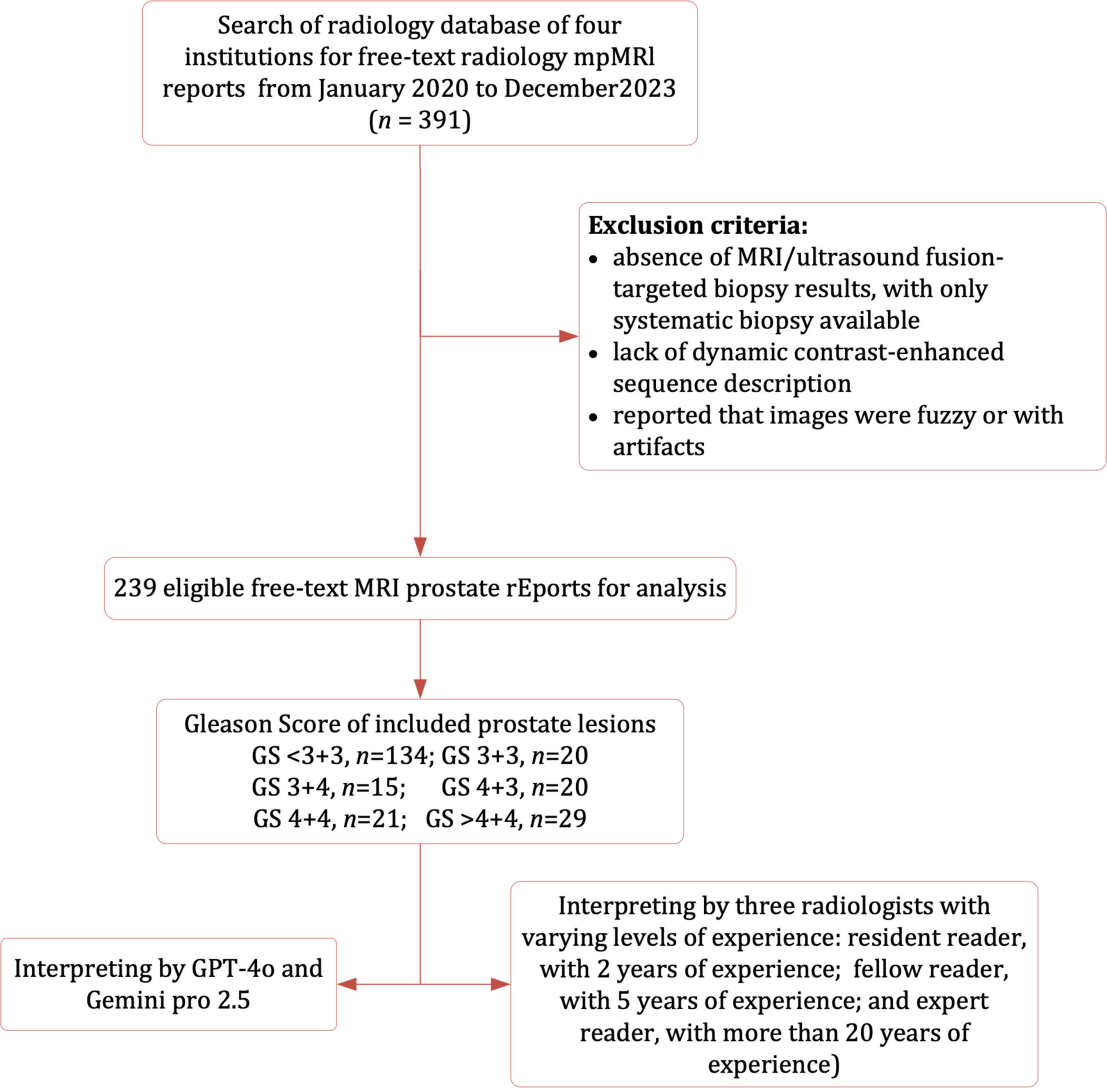
Flowchart of the study population with the exclusion criteria.

This exploratory retrospective study was approved by our institutional review board (IRB), which also waived the requirement for written informed consent. This study was conducted in accordance with the Declaration of Helsinki, and all data were collected in compliance with the Health Insurance Portability and Accountability Act (HIPAA). We retrieved the prostate MRI reports from our institutional radiology database between January 2021 and December 2024. All MRI prostate reports were generated by board-certified radiologists and written in free-text format. The search generated 391 MRI reports initially, of which 152 results were excluded because of the following reasons: 1) absence of MRI/ultrasound fusion-targeted biopsy results, with only systematic biopsy available; 2) lack of dynamic contrast-enhanced (DCE) sequence description; 3) reported that images were fuzzy or with artifacts. Finally, a total of 239 mpMRI reports were retained for analysis in our study (**Figure 1**). To compare the LLMs and human radiologists, three readers with varying levels of experience(resident reader, with 2 years of experience; fellow reader, with 5 years of experience; and expert reader, with more than 20 years of experience) reviewed these reports independently. Each radiologist assigned PI-RADS v2.1 categories to the dominant lesion, blinded to the original score, other parts of the report, and any patient-related data.

### PI-RADS category assignment by LLMs

All free-text prostate MRI reports were submitted to GPT (GPT-4o, March 2024 version) and Gemini (Gemini-2.5-pro) by using their respective application programming interface (API) (17, 18). These models offer enhanced multilingual support and robust API integration. Python (version 3.12.9) scripts were developed to automate report extraction from spreadsheets and submission to the LLMs. LangChain (version 0.3.25) was utilized as the backend framework to orchestrate interactions with the large language model via a script-based API, enabling standardized prompt construction and automated request handling. Pandas (version 0.3.25) was employed for structured data management, including the organization, indexing, and batch submission of report texts. The original radiology reports were submitted to the model in their raw form, without any manual or algorithmic preprocessing, to preserve the completeness and authenticity of the clinical language. The scoring outputs generated by the model were automatically retrieved through LangChain, parsed into structured tabular formats, and programmatically written to Microsoft Excel files using a fully automated pipeline. This end-to-end automation minimized human intervention and effectively reduced the risk of errors associated with manual operations, such as copying and pasting, thereby improving the reliability and reproducibility of the data collection process.

### Large language model evaluation

A standardized prompt template was developed to instruct the model to read each free-text report and extract or infer the most appropriate PI-RADS score according to the PI-RADS v2.1 criteria. The prompt included task clarification and guidance are presented in **Appendix S1**, and an example of original free-text prostate MRI report is demonstrated in **Appendix S2**. Each report was submitted to the model individually, and outputs were recorded. No fine-tuning or external training on study data was performed. For prompts for both LLMs, the temperature parameter was set to 0, which represented the randomness of the output, with lower values yielding more deterministic responses. All other model parameters were left at their default values. Subgroup analyses were conducted to examine the performance of LLMs according to prostate anatomy of the peripheral zone (PZ) and transitional zone (TZ).

### Statistical analysis

Due to the multi-category nature of the PI-RADS score and known limitations of Cohen’s kappa in this context, inter-rater agreement was assessed using Gwet’s AC1 coefficient, with 95% confidence intervals (CI). The following interpretive scale was used: 0-0.20 (slight agreement), 0.21-0.40 (fair), 0.41-0.60 (moderate), 0.61-0.80 (substantial), and 0.81-1.00 (almost perfect) (19, 20). Agreements were calculated among three human readers and between each human and two LLMs. Diagnostic performance metrics including sensitivity, specificity, and AUC were calculated using csPCa (defined as Gleason score ≥7) diagnosis from targeted biopsy as the reference standard. Differences in discordant assessments were evaluated using McNemar’s test, and overall diagnostic performance was compared using DeLong’s test. Statistical analyses were performed using STATA (version 18.1, StataCorp) and R (version 4.3.2), and a P-value <0.05 was considered statistically significant.

## Results

### Characteristics of patients and radiology reports

**Table 1** summarizes the characteristics of the 239 patients. The mean patient age was 71.34 ± 8.22 years. Based on MRI/US fusion targeted biopsy, csPCa was found in 85 patients (35.56%), whereas non-csPCa or PBH was found in 154 patients (64.44%). Of these lesions, 15 were categorized as Gleason score (GS) 3 + 4, 20 were GS 4 + 3, 21 were GS 4 + 4, and 29 were GS >4 + 4. Free-text style MRI prostate reports were generated by 5 different radiologists with 5–25 years of experience.

**Table 1:** Characteristics of Patients.

Characteristic	Value
Age (Years, mean±SD)	71.34±8.22
PSA (ng/mL, median [IQR])	11.33 (6.85-21.4)
PV (ml, median [IQR])	52.00 (36.40-72.38)
PSAD (ng/mL/mL, median [IQR])	0.21 (0.12-0.49)
Gleason score	
<3+3	134
3+3	20
3+4	15
4+3	20
4+4	21
>4+4	29

IQR, interquartile range; PSA, prostate-specific antigen; PSAD, prostate-specific antigen density; PV, prostate volume; SD, standard deviation.

### Agreement between human readers and LLMs

**Figure 2:**
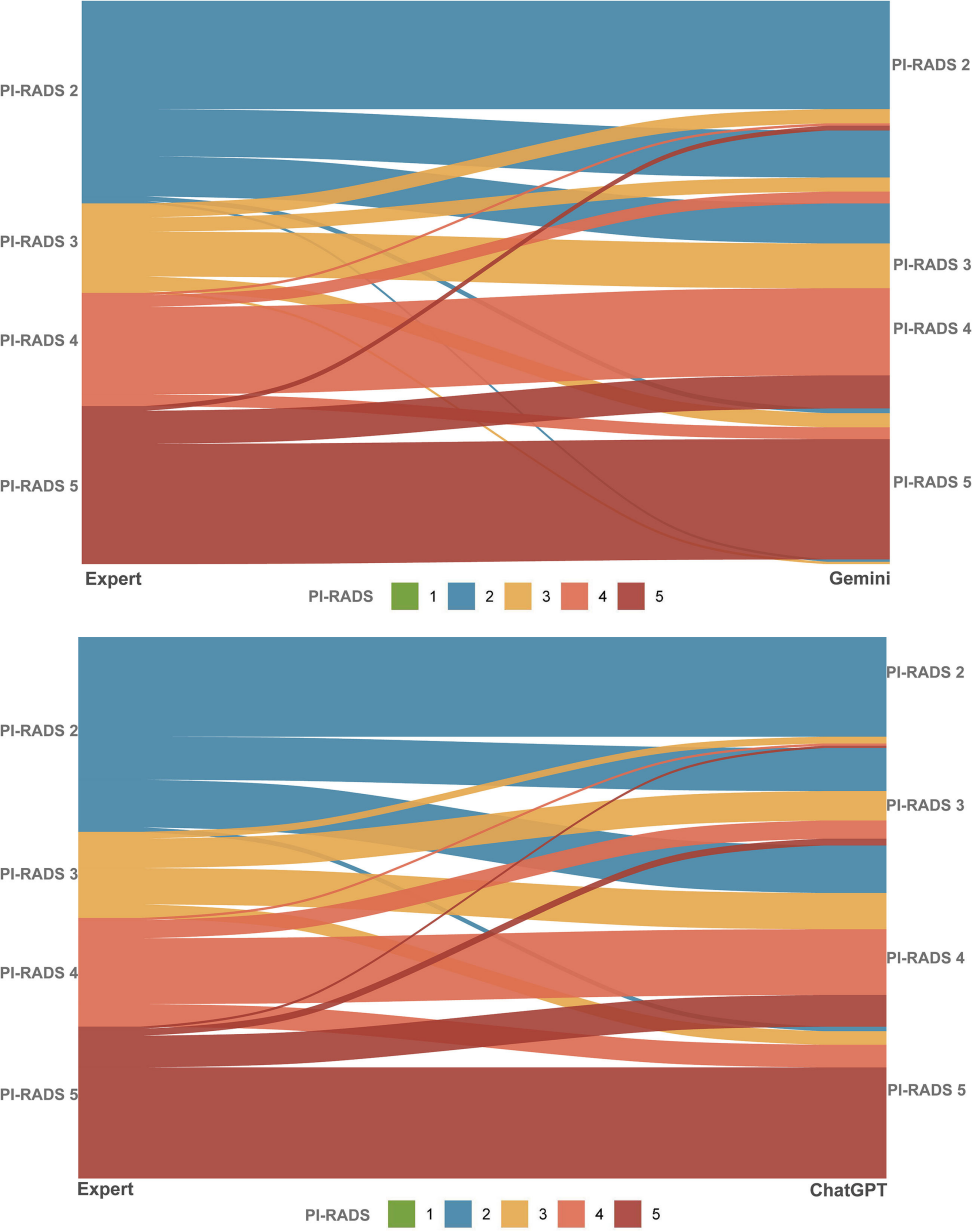
Sankey plots showing changes in PI-RADS category between human expert reader and LLMs. A, human expert reader-Gemini pro 2.5; B, human expert reader-GPT-4o.

The agreements between three radiologists with varied experience are presented in **Table 2**. The Gwet’s AC1 between expert and fellow readers was 0.68 (95% CI 0.61-0.75), between expert and resident was 0.50 (95% CI 0.42-0.58), and between fellow and resident was 0.50 (95% CI 0.42-0.58). By comparison, the agreement for LLMs between GPT and Gemini was 0.52 (95% CI 0.44-0.59), similar to those of among humans. We calculated the agreement between human readers and two LLMs, and the analysis showed that Gwet’s AC1 was slightly higher between radiologists and Gemini than GPT. For expert reader, Gwet’s AC1 was 0.49 (95% CI 0.41-0.57) for Gemini and 0.42 (95% CI 0.34-0.51, *P* = 0.17) for GPT. Whereas for fellow reader, the Gwet’s AC1 was 0.32 (95% CI 0.24-0.40) for Gemini and 0.31 (95% CI 0.23-0.38, *P* = 0.68) for GPT. **Table 3** shows the distribution of PI-RADS for human readers and LLMs, and **Figure 2** shows the changes in PI-RADS score assigned by radiologists and LLMs.

**Table 2:** Agreement for PI-RADS category 1–5 assignments between human readers and between human readers and LLMs.

Comparison	Percentage agreement (95% CI)	Gwet’s AC1 (95% CI)	*P* value
Expert vs. Fellow	0.74 (0.68-0.79)	0.68 (0.61-0.75)	Ref
Gemini 2.5 vs. GPT-4o	0.61 (0.54-0.67)	0.52 (0.44-0.59)	0.004
Expert vs. GPT-4o	0.56 (0.50-0.63)	0.42 (0.34-0.51)	<0.001
Fellow vs. GPT-4o	0.44 (0.37-0.50)	0.31 (0.23-0.38)	<0.001
Expert vs. Gemini 2.5	0.59 (0.52-0.65)	0.49 (0.41-0.57)	<0.001
Fellow vs. Gemini 2.5	0.45 (0.38-0.51)	0.32 (0.24-0.40)	<0.001

PI-RADS, Prostate Imaging Reporting and Data System.

**Table 3:** Distribution of PI-RADS for human readers and LLMs.

PI-RADS	Resident	Fellow	Expert	Gemini 2.5	GPT-4o
1	/	3/10	/	0/2	/
2	11/107	2/94	1/86	2/55	0/49
3	13/39	6/33	7/38	3/31	7/43
4	18/37	41/59	21/48	29/87	23/80
5	43/56	33/43	56/67	51/64	54/67

Data are presented with malignant/total.

LLM, Large language models; PI-RADS, Prostate Imaging Reporting and Data System.

For the subgroup of PZ, Gwet’s AC1 between expert and fellow readers was 0.79 (95% CI 0.71-0.88), and between Gemini and GPT was 0.64 (95% CI 0.54-0.74), respectively. Concerning inter-rater agreement between human readers and LLMs, the Gwet’s AC1 for expert reader were 0.59 (95% CI 0.48-0.69) for Gemini and 0.50 (95% CI 0.39-0.62) for GPT, and for fellow reader were 0.35 (95% CI 0.24-0.47) for Gemini and 0.39 (95% CI 0.28-0.50) for GPT. Details are presented in **Supplementary Table 1**. For TZ, the Gwet’s AC1 between expert and fellow reader was 0.56 (95% CI 0.46-0.67), and between Gemini and GPT was 0.37 (95% CI 0.25-0.49), which was lower than PZ. The agreement between the expert reader and two LLMs was comparable (Gwet’s AC1 0.37, 95% CI 0.24-0.49 for GPT and 0.37, 95% CI 0.25-0.49 for Gemini). However, the agreement between fellow reader and Gemini was significantly higher than GPT, with Gwet’s AC1 0.31 (95% CI 0.20-0.40) vs. 0.23 (95% CI 0.12-0.34).

### Agreement according to clinical management

As no PI-RADS score 1 was assigned by the resident and expert readers, and GPT-4o, the PI-RADS categories were grouped based on clinical management categories, namely, PI-RADS 1-2 (likely benign), PI-RADS 3 (indeterminate), and PI-RADS 4-5. Compared with human readers, LLMs categorized more lesions as malignant (PI-RADS 4-5, 151 in Gemini and 148 in GPT). The inter-reader agreement was not improved between expert and fellow radiologists; however, the agreement was improved significantly between Gemini and GPT (Gwet’s AC1 0.65, 95% CI 0.57-0.73, P<0.001). Other Gwet’s AC1 values were higher as compared with PI-RADS 1–5 classification, as shown in **Supplementary Table 2**. Among the 42 lesions categorized as PI-RADS 2 by the expert reader, nearly half (19 lesions) were classified as PI-RADS 3 by ChatGPT. Of the remaining 23 lesions that were misclassified as PI-RADS 4 (21 lesions) or PI-RADS 5 (2 lesions), the primary reason may have been vague descriptions of the TZ and PZ, which prevented the LLM from determining the dominant MRI sequence. Among the 25 lesions assigned a PI-RADS 3 score by the expert reader, 22 were upgraded by ChatGPT to PI-RADS 4 (16 lesions) or PI-RADS 5 (6 lesions), primarily due to the absence or ambiguity of DCE imaging descriptions. For Gemini, 19 of the 40 lesions categorized as PI-RADS 2 by the expert reader were upgraded to PI-RADS 4 (17 lesions) or PI-RADS 5 (2 lesions). Similar to ChatGPT, this misclassification was mainly attributable to vague descriptions of the TZ/PZ, which hindered correct sequence prioritization. Regarding PI-RADS 3 lesions, 25 of 32 were upgraded by Gemini to PI-RADS 4 (17 lesions) or PI-RADS 5 (8 lesions), again largely due to absent or insufficient DCE imaging information.

### Diagnostic performance of human readers and LLMs

**Figure 3:**
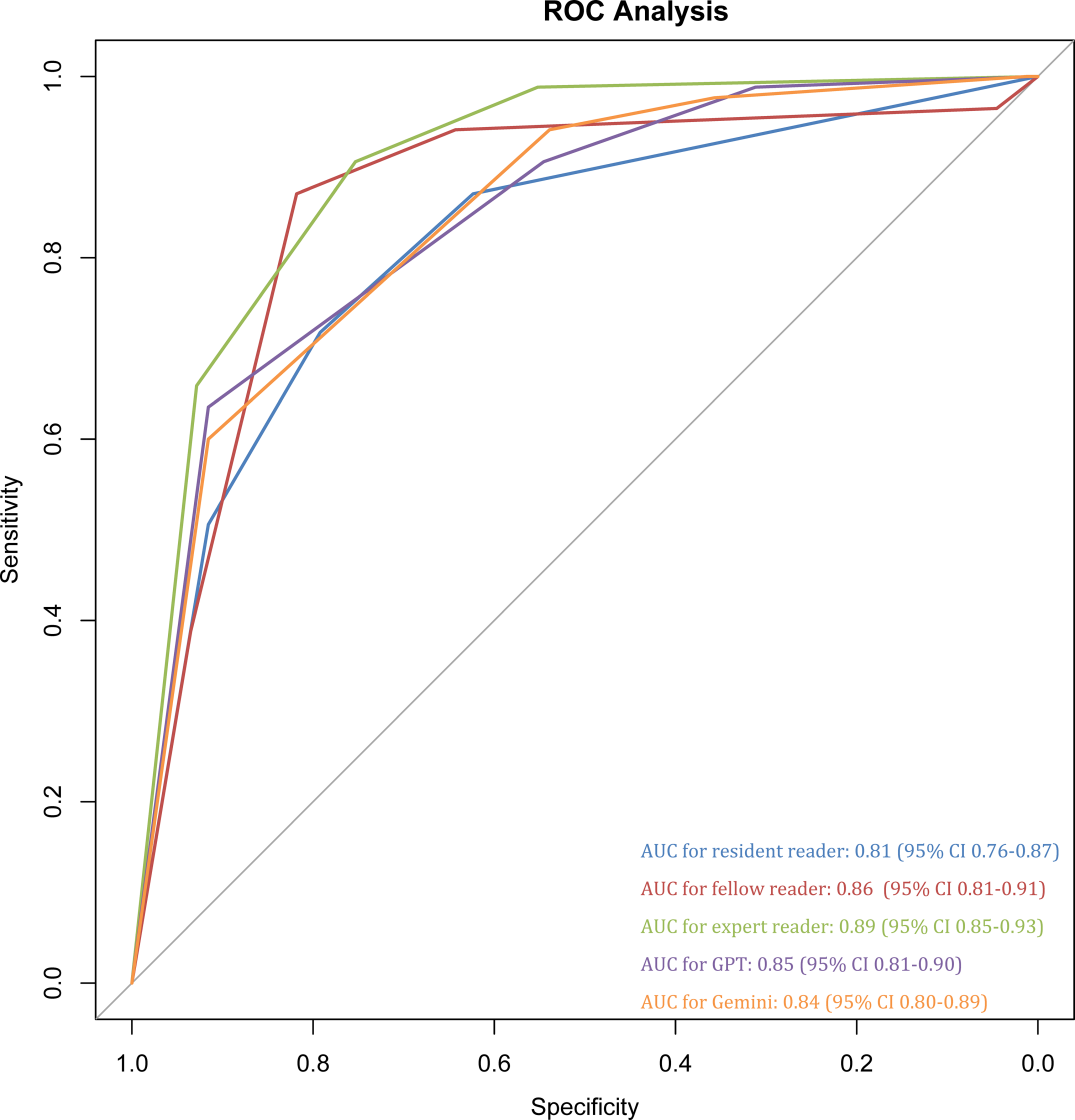
ROC analysis for prediction of clinically significant prostate cancer.

**Table 4** and **Figure 3** summarizes the performance of LLMs and human readers of the stratification of csPCa based on free-text MRI reports. GPT yielded an AUC of 0.85 (95% CI 0.81-0.90), higher than Gemini (0.84, 95% CI 0.80-0.89) and resident reader (0.81 95% CI 0.76-0.87), but lower than expert (0.89, 95% CI 0.85-0.93) and fellow readers (0.86, 95% CI 0.81-0.91). It should be noted that LLMs are prone to yielding high sensitivity but low specificity. As for human readers, only 93, 102, and 115 lesions were classified to PI-RADS 4–5 for resident, fellow, and expert radiologists. Both LLMs assigned a similar number of indeterminate lesions (PI-RADS 3) with human readers. There were respective 31 lesions and 43 lesions were categorized as PI-RADS score 3 by Gemini and GPT, and of them 3 and 7 lesions were csPCa.

**Table 4:** Diagnostic performance for each rater.

Rater	Cutoff	Sensitivity	Specificity	AUC (95% CI)	*P*
Resident	PI-RADS≥3	87.06%	62.75%	0.81 (0.76-0.87)	0.002
	PI-RADS≥4	71.76%	79.08%		
Fellow	PI-RADS≥3	94.12%	64.29%	0.86 (0.81-0.91)	0.06
	PI-RADS≥4	87.06%	81.82%		
Expert	PI-RADS≥3	98.82%	54.90%	0.89 (0.85-0.93)	reference
	PI-RADS≥4	90.59%	75.16%		
GPT-4o	PI-RADS≥3	100.00%	31.17%	0.85 (0.81-0.90)	0.05
	PI-RADS≥4	91.67%	54.55%		
Gemini-2.5	PI-RADS≥3	97.65%	35.71%	0.84 (0.80-0.89)	0.02
	PI-RADS≥4	94.12%	53.90%		

PI-RADS, Prostate Imaging Reporting and Data System.

## Discussion

This study evaluated the agreement between three human readers and two state-of-the-art LLMs in assigning PI-RADS v2.1 scores using free-text prostate MRI reports, along with their diagnostic performance. Our results demonstrated that compared with substantial agreement between experienced human readers (expert-fellow, Gwet’s AC1 = 0.68), GPT and Gemini showed only moderate agreement, with Gwet’s AC1 = 0.52, significantly lower than human readers (P = 0.004). Moreover, the agreement between LLMs and expert reader (Gwet’s AC1 = 0.42-0.49) were significantly higher than between LLMs and fellow readers (Gwet’s AC1 = 0.31-0.32; P<0.001). When PI-RADS scores were grouped into clinically relevant management strata, categories 1-2 (likely benign), 3 (indeterminate), and 4-5 (suspicious for csPCa), the agreement between LLMs was improved significantly. For GPT, the Gwet’s AC1 was increased to 0.53 and 0.45 for expert and fellow reader, then for Gemini were 0.54 (expert reader) and 0.45 (fellow reader). Additionally, the agreement between two LLMs increased to Gwet’s AC1 0.64. As for diagnostic performance, the expert radiologist achieved the highest AUC of 0.89, followed by the fellow (AUC 0.86) and the resident (AUC 0.81). Interestingly, both GPT-4o (AUC 0.85, 95% CI: 0.81–0.90) and Gemini (AUC 0.84, 95% CI: 0.80–0.89) performed comparably to the fellow-level diagnostic performance. However, LLMs consistently demonstrated higher sensitivity, 100% for GPT-4o and 97.65% for Gemini using a PI-RADS ≥3 threshold, but at the cost of significantly lower specificity (31.17% for GPT-4o and 35.71% for Gemini), indicating a tendency to overestimate malignancy risk. In a recent study, Lee et al. assessed the performance of several LLMs against radiologists for PI-RADS classification with prostate mpMRI reports. They found GTP-4o mini and Gemini-1.5 demonstrated almost perfect inter-reader agreement (Kappa=0.86 and 0.81) with the original PI-RADS score, higher than our results (21).

In our study, the low agreement between resident and expert radiologists (Gwet’s AC1 = 0.51) highlighted the steep learning curve in accurate PI-RADS interpretation. The fact that GPT and Gemini achieved similar or slightly better agreement than less experienced readers suggested that LLMs may help reduce variability among novice and general radiologists in complex decision-making tasks (10, 22). However, it is also important to emphasize that neither LLM matched the accuracy or discriminative ability of the expert reader, particularly when evaluated by AUC (P = 0.05 for GPT and P = 0.02 for Gemini). The discrepancy in performance across anatomical zones further emphasizes the complexity of the task. Inter-rater agreement among both human and machine readers was significantly higher for lesions located in the PZ compared to the TZ. This is consistent with prior studies reporting greater interpretive difficulty and lower interobserver agreement in TZ lesions due to their variable appearance and overlap with benign prostatic hyperplasia (5, 6). The relatively high agreement in the PZ (Gwet’s AC1 = 0.82 between expert and fellow) likely reflects the clearer imaging characteristics and more deterministic rules applied in the PI-RADS v2.1 framework for this zone. Conversely, the lower agreement in TZ, especially between LLMs (Gwet’s AC1 = 0.37), indicates that models may struggle with subtle interpretive distinctions in narrative descriptions, such as stromal versus glandular composition, presence of pseudocapsule, or signal heterogeneity, which are often implicit or ambiguously described in reports (23, 24). Considering that LLM outputs may not be strictly deterministic even when the temperature parameter is set to zero, we implemented a fixed and predefined prompt and applied deterministic decoding settings across all experiments. Furthermore, we conducted repeated inference runs for each report (multiple independent runs per report) to assess output stability. The resulting PI-RADS scores demonstrated a high level of consistency across runs, with negligible variability and no impact on the final classification or statistical analyses. These results indicated that the LLM-generated PI-RADS assessments were robust and reproducible under the specified experimental conditions. An important consideration in this study is the reliance on free-text radiology reports as the sole input for LLMs. While narrative reports inevitably reflect the reporting radiologist’s experience, style, and level of detail, such heterogeneity is not a limitation unique to this study but rather a defining characteristic of real-world clinical practice. Despite the widespread promotion of structured PI-RADS templates, free-text reporting remains prevalent across institutions, and variability in report quality is a well-recognized contributor to inter-reader disagreement in PI-RADS assessment.

In addition to diagnosis and management, the potential utility of LLMs may be further enhanced by integrating clinical data beyond imaging reports alone (10, 25, 26). Prostate cancer risk stratification and management decisions often require consideration of multiple variables, including serum PSA levels, PSA density, digital rectal examination findings, prostate volume, prior biopsy history, and family or genetic risk factors. By incorporating clinical parameters, LLMs may provide more holistic clinical reasoning. Recent advances in radiology foundation models capable of processing imaging data directly such as RadFM, MRICore, and MedGemma further highlight the growing interest in multimodal medical AI systems (27–29). These models primarily focus on image-level representation learning and multimodal diagnostic modeling, whereas the present study addresses a complementary but distinct clinical scenario: report-level language understanding and inference from free-text prostate MRI reports, which remain prevalent in routine practice. From this perspective, text-based LLMs and imaging-centric foundation models should be viewed as synergistic rather than competing approaches. In decision-making scenarios, LLMs could serve as intermediaries to synthesize clinical, laboratory, and imaging data into structured summaries or personalized risk profiles for both clinicians and patients (30, 31). This multimodal integration supports the growing trend toward precision medicine, where treatment pathways can be customized for individual patients instead of depending exclusively on imaging-based evaluations. While our current study focused on free-text MRI report interpretation, future work should explore the role of LLMs in multimodal data fusion to support end-to-end clinical decision-making in prostate cancer care.

There are several limitations to our study. First, the retrospective design and single-institution dataset may limit the generalizability of findings, particularly as institutional reporting styles and wording conventions can vary. Second, the models were not fine-tuned on radiologic data nor trained explicitly on PI-RADS-specific corpora. While this reflects real-world constraints and the performance of off-the-shelf tools, domain-specific adaptation could potentially yield improved accuracy and consistency. Third, our approach relied solely on textual information and did not incorporate image-level data. Although several radiology foundation models have recently been proposed to process imaging data directly, these models differ fundamentally from general-purpose large language models in terms of design objectives, data requirements, and clinical deployment. The present study therefore focused on text-based LLMs to address report-level interpretation, which remains a common and clinically relevant scenario. Future work may benefit from integrating multimodal LLMs capable of processing both imaging and textual inputs, potentially narrowing the performance gap between machines and expert radiologists. Lastly, our study focused exclusively on prostate MRI reports, which may limit the generalizability of our findings to other linguistic contexts. Radiology reporting styles, structure, and terminology can differ substantially between languages and regions, which may affect model behavior and interpretive accuracy.

## Conclusion

LLMs demonstrated promising performance in assigning PI-RADS scores from free-text prostate MRI reports, with accuracy and agreement approaching that of general radiologists. These findings support its potential as a supplementary tool for report standardization and trainee education. However, both LLMs demonstrated a tendency to overestimate malignancy risk, with higher proportions of lesions assigned to PI-RADS categories 4 and 5 compared to human readers. While these models show promise as assistive tools, they are not yet ready to replace expert interpretation in high-stakes clinical settings.

## Data Availability

The raw data supporting the conclusions of this article will be made available by the authors, without undue reservation.
