# Supplementary material for: Evaluation of large language models for PI-RADS score extraction from free-text prostate MRI reports: a comparative study with human readers
Source: Front Oncol. 2026 Apr 10;16:1743096. doi: 10.3389/fonc.2026.1743096 (PMC13107145; doi:10.3389/fonc.2026.1743096)
Supplement: Supplementary file 1 [file DataSheet1.docx]

**Appendix S1**. LLM Prompts used in this study are as follows:

You are an experienced genitourinary radiologist with extensive expertise in prostate MRI and the PI-RADS v2.1 scoring system. Based on the following prostate MRI radiology report, please analyze and score any suspicious lesion(s) strictly in accordance with the PI-RADS v2.1 criteria.

**Task Requirements:**

1. Determine whether any suspicious prostate lesion is present;
2. Specify the anatomical zone of the lesion (Peripheral Zone or Transition Zone);
3. Perform sequence-based assessment according to the dominant sequence:
   - Peripheral Zone: DWI as the dominant sequence;
   - Transition Zone: T2-weighted imaging (T2WI) as the dominant sequence;
4. Integrate findings from DWI, ADC, T2WI, and dynamic contrast-enhanced (DCE) imaging (if description for some sequences is missing, regarded as normal);
5. Assign a PI-RADS v2.1 score (1–5) to each suspicious lesion;
6. If multiple lesions are present, select only the highest-scoring lesion as the index lesion;
7. If no definite suspicious lesion is identified, explain the reasons and assign a PI-RADS score of 1 or 2.

**Output Format Requirements** (Please strictly follow the structured format below):

1. Number of lesions: __
2. Index lesion location:
   - Zone (PZ / TZ):
3. Imaging characteristics by sequence:
   - T2WI:
   - DWI:
   - ADC:
   - DCE:
4. Rationale for dominant sequence selection:
5. Integrated assessment:
6. Final PI-RADS v2.1 score: __

**Appendix S2. An example of our prostate MRI report:**

The prostate gland measures approximately 61 mm (left–right) × 56 mm (anteroposterior) × 75 mm (craniocaudal). The transition zone is enlarged with heterogeneous signal intensity, containing multiple nodular lesions. These nodules demonstrate mixed hyperintense and hypointense signals on T2WI. At the prostatic base, a nodular lesion is noted, showing hypointensity on T2WI, hyperintensity on DWI, and hypointensity on the ADC map. The hyperplastic transition zone protrudes superiorly into the bladder, resulting in significant compression of the urinary bladder. The peripheral zone is compressed and thinned, with heterogeneous signal intensity. The boundary between the transition zone and the peripheral zone is clearly defined. The prostatic capsule appears smooth and intact. The bilateral seminal vesicles are normal in size and morphology, with no abnormal signal intensity detected.

The bladder is suboptimally distended, and the bladder wall shows no focal thickening. The rectal wall is smooth, without focal thickening, and the perirectal fat planes are preserved. No enlarged lymph nodes are identified in the bilateral inguinal regions or along the iliac vessels. No pelvic free fluid is observed. The visualized pelvic bones show no abnormal signal intensity. DCE images demonstrates marked heterogeneous enhancement of the prostate gland. No abnormal enhancement is observed in the remaining pelvic bones or soft tissues.
