# Supplementary material for: Evaluation of large language models for PI-RADS score extraction from free-text prostate MRI reports: a comparative study with human readers
Source: Front Oncol. 2026 Apr 10;16:1743096. doi: 10.3389/fonc.2026.1743096 (PMC13107145; doi:10.3389/fonc.2026.1743096)
Supplement: Supplementary file 2 [file Table1.docx]

**Table S1**. Agreement for PI-RADS Category 1–5 Assignments between Raters According to Zone Anatomy

| **Comparison** | **Percentage Agreement (95% CI)** | **Gwet’s AC1**  **(95% CI)** |
| --- | --- | --- |
| **PZ** |  |  |
| Expert vs. Fellow | 0.83 (0.76-0.89) | 0.79 (0.71-0.88) |
| Gemini 2.5 vs. GPT-4o | 0.70 (0.62-0.79) | 0.64 (0.54-0.74) |
| Expert vs. GPT-4o | 0.62 (0.53-0.71) | 0.50 (0.39-0.62) |
| Fellow vs. GPT-4o | 0.50 (0.41-0.59) | 0.39 (0.28-0.50) |
| Expert vs. Gemini 2.5 | 0.66 (0.57-0.75) | 0.59 (0.48-0.69) |
| Fellow vs. Gemini 2.5 | 0.43 (0.34-0.52) | 0.35 (0.24-0.47) |
| **TZ** | | |
| Expert vs. Fellow | 0.64 (0.56-0.73) | 0.56 (0.46-0.67) |
| Gemini 2.5 vs. GPT-4o | 0.52 (0.43-0.61) | 0.37 (0.25-0.49) |
| Expert vs. GPT-4o | 0.51 (0.42-0.60) | 0.37 (0.24-0.49) |
| Fellow vs. GPT-4o | 0.37 (0.28-0.46) | 0.23 (0.12-0.34) |
| Expert vs. Gemini 2.5 | 0.51 (0.42-0.60) | 0.37 (0.25-0.49) |
| Fellow vs. Gemini 2.5 | 0.46 (0.37-0.55) | 0.31 (0.20-0.42) |

**Table S2**. Agreement for Grouped PI-RADS Category Raters

| **Comparison** | **Percentage Agreement (95% CI)** | **Gwet’s AC1**  **(95% CI)** |
| --- | --- | --- |
| Expert vs. Fellow | 0.85 (0.81-0.89) | 0.79 (0.72-0.85) |
| Gemini 2.5 vs. GPT-4o | 0.74 (0.68-0.80) | 0.65 (0.56-0.73) |
| Expert vs. GPT-4o | 0.67 (0.61-0.73) | 0.53 (0.44-0.62) |
| Fellow vs. GPT-4o | 0.62 (0.55-0.68) | 0.45 (0.36-0.54) |
| Expert vs. Gemini 2.5 | 0.67 (0.61-0.73) | 0.54 (0.45-0.63) |
| Fellow vs. Gemini 2.5 | 0.61 (0.55-0.67) | 0.45 (0.35-0.54) |
